# A Novel Modification of PSO Algorithm for SML Estimation of DOA

**DOI:** 10.3390/s16122188

**Published:** 2016-12-19

**Authors:** Haihua Chen, Shibao Li, Jianhang Liu, Fen Liu, Masakiyo Suzuki

**Affiliations:** 1 College of Computer and Communication Engineering, China University of Petroleum, Qingdao 266580, China; lishibao@upc.edu.cn (S.L.); liujianhang@upc.edu.cn (J.L.); lfupc2014@163.com (F.L.); 2 Graduate School of Engineering, Kitami Institute of Technology, Kitami 090-8507, Japan; masakiyo@mail.kitami-it.ac.jp

**Keywords:** direction-of-arrival, stochastic maximum likelihood, Particle Swarm Optimization (PSO) algorithm, computational complexity

## Abstract

This paper addresses the issue of reducing the computational complexity of Stochastic Maximum Likelihood (SML) estimation of Direction-of-Arrival (DOA). The SML algorithm is well-known for its high accuracy of DOA estimation in sensor array signal processing. However, its computational complexity is very high because the estimation of SML criteria is a multi-dimensional non-linear optimization problem. As a result, it is hard to apply the SML algorithm to real systems. The Particle Swarm Optimization (PSO) algorithm is considered as a rather efficient method for multi-dimensional non-linear optimization problems in DOA estimation. However, the conventional PSO algorithm suffers two defects, namely, too many particles and too many iteration times. Therefore, the computational complexity of SML estimation using conventional PSO algorithm is still a little high. To overcome these two defects and to reduce computational complexity further, this paper proposes a novel modification of the conventional PSO algorithm for SML estimation and we call it Joint-PSO algorithm. The core idea of the modification lies in that it uses the solution of Estimation of Signal Parameters via Rotational Invariance Techniques (ESPRIT) and stochastic Cramer-Rao bound (CRB) to determine a novel initialization space. Since this initialization space is already close to the solution of SML, fewer particles and fewer iteration times are needed. As a result, the computational complexity can be greatly reduced. In simulation, we compare the proposed algorithm with the conventional PSO algorithm, the classic Altering Minimization (AM) algorithm and Genetic algorithm (GA). Simulation results show that our proposed algorithm is one of the most efficient solving algorithms and it shows great potential for the application of SML in real systems.

## 1. Introduction

The localization of multiple signal sources by a passive sensor array is of great importance in a wide variety of fields, such as radar, communications, underwater acoustics and so on. The basic problem in this context is to estimate directions-of-arrival (DOA) of narrow-band signal sources located in the far field of the array.

For the DOA estimation problem, now there are mainly three types of solving techniques. The first type is the “Subspace Decomposition Based Algorithm” such as Multiple Signal Classification (MUSIC) [1,2] and Estimation of Signal Parameters via Rotational Invariance Techniques (ESPRIT) [3]. This type is the most widely used in practical systems [4,5] at present because its accuracy is acceptable in some cases and its computational complexity is low enough so that timeliness of the system can be guaranteed. In particular the ESPRIT can calculate the DOA explicitly. Therefore, its computational complexity is extremely low. However, these two algorithms cannot deal with coherent signals directly which may happen for example in multi-path propagations in real environment. In this case pre-processing techniques such as spatial smoothing [6] and matrix reconstruction [7] methods are needed. Furthermore, the accuracy of DOA estimation of MUSIC and ESPRIT decreases greatly when Signal-to-Noise Ratio (SNR) gets lower for both coherent and non-coherent signals cases.

The second type is the “Subspace Fitting Based Algorithm”. The representative algorithms are Deterministic Maximum Likelihood (DML) [8], Stochastic Maximum Likelihood (SML) [9,10] and Weighted Subspace Fitting (WSF) [11]. These algorithms can deal with small number of snapshots. They can also handle coherent signals without any pre-processing technique and the accuracy of DOA estimation is also much higher than that of MUSIC and ESPRIT. Therefore, this type is much more superior to the first type. However, the disadvantage of this type is that the criteria of DML, SML and WSF are multi-dimensional non-linear optimization problems. As a result, the computational complexity of this type is usually very high and it is hard to be applied to real systems.

The third type is the latest “compressed sensing techniques” [12,13]. This technique is based on the principle that, through optimization, the sparsity of a signal can be exploited to recover it from far fewer samples than required by the Shannon-Nyquist sampling theorem. The feature of this kind of algorithm is that it needs fewer samples, but the accuracy of DOA estimation is also much lower than that of the second type.

Among all these techniques, the SML algorithm is well-known for its high accuracy of DOA estimation [14,15,16]. The only drawback of SML is that the computational complexity is too high (the estimation of SML criteria is a multi-dimensional non-linear optimization problem) to be applied to real systems. Therefore, this paper addresses the issue of reducing the computational complexity of SML estimation of DOA.

To solve the multi-dimensional non-linear optimization problem of SML estimation, there are some classic solving algorithms such as Alternating Minimization (AM) algorithm [9], Genetic Algorithm (GA) [17], ant colony algorithm [18] and Particle Swarm Optimization (PSO) algorithm [19,20]. Especially, the PSO algorithm is considered as a rather efficient method for multi-dimensional non-linear optimization problems in DOA estimation.

The PSO algorithm is a population-based search algorithm based on the simulation of a social behavior of birds within a flock. Due to the moving mechanism of particles (individuals of birds), the closer the particles are to the optimal value (“food”), the faster all the particles converge. Since the whole search space is large, this algorithm usually needs a large number of particles (randomly initiated in the whole search space), and the total iteration times are also a little high when all the particles converge. Therefore, the computational complexity of the PSO algorithm is also a little high.

In this paper, we propose a novel modification of the conventional PSO algorithm for SML estimation and we call it Joint-PSO algorithm. This new algorithm can overcome the two defects mentioned above, i.e., too many particles and too many iterations. Firstly, we use the solution of ESPRIT and Cramer-Rao bound (CRB) to determine a “small area” in the search space. Since the solution of ESPRIT (also note that the computational complexity of calculating ESPRIT is extremely low) can be considered to be near the solution of SML, that “small area” can also be considered to be very close to the solution of SML. Therefore, fewer particles are needed to be initiated in that small area. Since these particles are already very close to the solution of SML, they will converge quickly. Then the iteration times can also be greatly reduced. As a result, the computational complexity can be greatly reduced compared to that of the original PSO algorithm.

In simulation, we compare the proposed algorithm with the conventional PSO algorithm, the classic Altering Minimization (AM) algorithm and Genetic algorithm (GA). Simulation results show that our proposed algorithm is one of the most efficient solving algorithms and it shows great potential for the application of SML in real systems.

The rest of this paper is organized as follows. In Section 2 we introduce the problem of DOA and the formulation of SML. In Section 3, we show the conventional PSO algorithm and the proposed Joint-PSO algorithm. Simulation results are shown in Section 4 and conclusion is drawn in Section 5.

## 2. System Model and Problem Formulations

Without loss of generality, consider that there are *p* sensors and *q* narrow-band sources far from the array, centered around a known frequency, impinging on the sensor array from distinct directions $\theta_{1}$, $\theta_{2}$, ..., $\theta_{q}$, with respect to a reference point respectively.

Note that the received signals may be coherent because of multipath propagation. In the case where there are coherent signals, the independent signal number is less than *q*. The task of this paper is to detect all *q* directions. Also note that, here we assume that the signals are narrow-band. For wideband signals, the CSM algorithms [21] can be used as a pre-processing technique. Furthermore, the sensor configuration can be arbitrary [9] and we assume that all the sensors are omnidirectional and not coupled.

### 2.1. Problem Formulation of DOA

In this subsection, we show the basic problem formulation of DOA [9].

Using complex envelope representation, the *p*-dimensional vector received by the array can be expressed as

(1) $$x\left(t\right)=\sum_{k=1}^{q}a\left(\theta_{k}\right)s_{k}\left(t\right)+n\left(t\right),$$

where $s_{k}\left(t\right)$ is the *k*-th signal received at a certain reference point. n(t) is a *p*-dimensional noise vector. a(θ) is the “steering vector” of the array towards direction *θ*, which is presented as

(2) $$a\left(\theta \right)={\left[a_{1}\left(\theta \right)e^{-j\omega_{0}\tau_{1}\left(\theta \right)},\ldots ,a_{p}\left(\theta \right)e^{-j\omega_{0}\tau_{p}\left(\theta \right)}\right]}^{T}$$

where $a_{i}\left(\theta \right)$ is the amplitude response of the *i*-th sensor to a wave-front impinging from the direction *θ*. $\tau_{i}\left(\theta \right)$ is the propagation delay between the *i*-th sensor and the reference point. The superscript *T* denotes the transpose of a matrix.

In the matrix notation, Equation (1) can be rewritten as

(3) x(t)=A(Θ)s(t)+n(t),

(4) $$A\left(\Theta \right)=[\;\;a\left(\theta_{1}\right)\;\;a\left(\theta_{2}\right)\;\;\cdots \;\;a\left(\theta_{q}\right)\;\;],$$

(5) $$s\left(t\right)={\left[\;\;s_{1}\left(t\right)\;\;s_{2}\left(t\right)\;\;\cdots \;\;s_{q}\left(t\right)\;\;\right]}^{T},$$

(6) $$\Theta =\{\;\;\theta_{1}\;\;\theta_{2}\;\;\cdots \;\;\theta_{q}\;\;\}.$$

Suppose that the received vectors x(t) are sampled at *N* time instants (snapshots) $t_{1}$, $t_{2}$, ..., $t_{N}$ and define the matrix of the sampled data as

(7) $$X=[\;\;x\left(t_{1}\right)\;\;x\left(t_{2}\right)\;\;\cdots \;\;x\left(t_{N}\right)\;\;].$$

The problem of DOA estimation is to be stated as follows. Given the sampled data ***X***, obtain a set of estimated directions

(8) $$\hat{\Theta }=\left\{\;\;{\hat{\theta }}_{1}\;\;{\hat{\theta }}_{2}\;\;\cdots \;\;{\hat{\theta }}_{q}\;\;\right\}.$$

of $\theta_{1}$, $\theta_{2}$, ..., $\theta_{q}$.

### 2.2. SML Criteria

According to [9,22], the SML criteria is derived based on the following assumptions.

**(A1)**  The array configuration is known and any *p* steering vectors for different *q* directions are linearly independent, i.e., the matrix A(Θ) has full rank.
**(A2)**  $n(t_{i})$ are statistically independent samples from a complex Gaussian random vector with zero mean and the covariance matrix $\sigma^{2}I_{p}$, where $I_{p}$ is a p×p identity matrix and $\sigma^{2}$ is an unknown real number.
**(A3)**  $s(t_{i})$ are independent samples from a complex Gaussian random vector which has zero mean and signal covariance matrix ***S*** and is independent of the noise ($s(t_{i})$ are independent of $n(t_{j})$ for any *i* and *j*). Define $S_{o}$ as the signal matrix,
  (9) $$S_{o}=\left[\;s\left(t_{1}\right)\;\;s\left(t_{2}\right)\;\;\cdots \;\;s\left(t_{N}\right)\;\right].$$
  The signal covariance matrix ***S*** is defined as
  $$S=E[S_{o}S_{o}^{H}],$$
  where E[ ] means expectation and rank[S]=r. In the case of r<q, the signals are coherent or fully correlated.
**(A4)**  q<p, p,q and *r* are known. The snapshots *N* should be greater than *r*.
**(A5)**  *p*, *q* and *r* satisfy the condition that a unique solution of DOA exists in the noise-free case. When the direction *θ* is expressed by a single real parameter, the sufficient condition of the uniqueness is given by q<2rp/(2r+1) and the necessary condition is given by q≤2rp/(2r+1) [23].

The SML criteria can be derived as follows.

(10) $${\hat{\Theta }}_{SML}=\arg\min_{\Theta }L_{\text{SML}}\left(\Theta \right),$$

(11) $$L_{\text{SML}}\left(\Theta \right)=\det R_{SS}\times {\left(\frac{1}{p-q}\text{tr}\left\{R_{NN}\right\}\right)}^{p-q},$$

(12) $$R_{NN}=V_{N}^{H}\left(\Theta \right)\hat{R}V_{N}\left(\Theta \right),$$

(13) $$R_{SS}=V_{S}^{H}\left(\Theta \right)\hat{R}V_{S}\left(\Theta \right),$$

where

(14) $$\hat{R}=\frac{1}{N}XX^{H},$$

is the sample covariance matrix of the sampled data. $V_{S}\left(\Theta \right)$ is a p×q matrix composed of an orthonormal system of the signal subspace spanned by A(Θ). $V_{N}\left(\Theta \right)$ is a p×(p−q) matrix composed of an orthonormal system of the noise subspace, which is an orthogonal complement of the signal subspace. In real calculation, $V_{S}\left(\Theta \right)$ and $V_{N}\left(\Theta \right)$ can be easily got by applying a QR decomposition to A(Θ). The q×q matrix, $R_{SS}$, corresponds to the covariance matrix of the components for x(t) in the signal subspace. $R_{NN}$ is the covariance matrix of the components for x(t) in the noise subspace.

Note that there are literatures [10,24] discussing about the rigor of SML criteria derived above. In [10], a constraint condition in which the estimated signal covariance matrix must be non-negative definite is imposed and a more complex criteria is derived. As discussed in literatures [10,24], the criteria of SML above, i.e., Equation (10) is still valid except for some rare cases for example for very low SNRs or very few snapshots. And also as we will show next that our proposed Joint-PSO algorithm is general, it can also be applied to the criteria proposed in [10]. Since this paper focuses on the problem of reducing the computational complexity of SML estimation and our proposed algorithm is general, the proposed algorithm is applied to the SML criteria (10).

## 3. PSO Algorithm for SML

The PSO algorithm is a population-based search algorithm based on the simulation of a social behavior of birds within a flock. In PSO, individuals, referred to as particles, are “flown” through hyper-dimensional search space. Changes to the positions of particles within the search space are based on the social-psychological tendency of individuals to emulate the success of other individuals. The consequence of modeling this social behavior is that the search process is such that particles stochastically return toward previously successful regions in the search space.

### 3.1. Conventional PSO for SML

The PSO algorithm can be applied for SML estimation as follows.

(1) Construct a swarm of particles $\bar{\Theta }=\left\{{\tilde{\Theta }}_{1},{\tilde{\Theta }}_{2},\ldots ,{\tilde{\Theta }}_{m}\right\}$ whose position might be the potential solution to the optimization problem. *m* is the number of particles. ${\tilde{\Theta }}_{i}=\left(\theta_{i1},\theta_{i2},\ldots ,\theta_{iq}\right)$ is defined as the *i*th (1≤i≤m) particle’s position in the search space. The initial position of each particle is random in the search space.

(2) Each particle uses the local best position found by itself and the global best position found by the swarm for updating. Let *i* represents the *i*-th particle; *l* represents the *l*-th iteration; ${\tilde{\Theta }}_{i}^{l}$ denotes the position of the *i*-th particle in the *l*-th iteration. Define $T_{i}^{l}$ as the local best position of the *i*-th particle which can find for *l* times of iteration. Obviously,

(15) $$T_{i}^{0}={\tilde{\Theta }}_{i}^{0},$$

(16) $$T_{i}^{1}=\arg\min_{{\tilde{\Theta }}_{i}^{0},{\tilde{\Theta }}_{i}^{1}}L_{SML},\ldots$$

(17) $$T_{i}^{l}=\arg\min_{{\tilde{\Theta }}_{i}^{0},\ldots ,{\tilde{\Theta }}_{i}^{l}}L_{SML},$$

where $L_{SML}$ is the criteria of SML which is defined in Equation (10). Define $G^{l}$ as the global best position of the whole swarm (*m* particles) which can find for *l* times iteration. Therefore,

(18) $$G^{l}=min\left\{T_{1}^{l},T_{2}^{l},\ldots ,T_{m}^{l}\right\}.$$

The moving (updating) of the *i*-th particle will be affected by both $T_{i}^{l}$ and $G^{l}$.

(3) The updating of the *i*-th particles is accomplished by the following equations:

(19) $${\tilde{\Theta }}_{i}^{l+1}={\tilde{\Theta }}_{i}^{l}+{\tilde{V}}_{i}^{l+1};$$

where,

(20) $${\tilde{V}}_{i}^{l+1}=w{\tilde{V}}_{i}^{l}+c_{1}r_{1}\left(T_{i}^{l}-{\tilde{\Theta }}_{i}^{l}\right)+c_{2}r_{2}\left(G^{l}-{\tilde{\Theta }}_{i}^{l}\right).$$

${\tilde{V}}_{i}^{l}$ is the moving speed of the *i*-th particle in the *l*-th iteration and ${\tilde{V}}_{i}^{0}=0.5.$ *l* is the updating time; *w* is the inertia factor; $c_{1}$ is self confidence factor; $c_{2}$ is swarm confidence factor; $r_{1}$ and $r_{2}$ are uniformly distributed random variables between 0 and 1. Generally, the value of *w* should be between 0.1 to 0.9 and is usually set to be 0.5; $c_{1}=c_{2}=2$. [19,20]

(4) Update velocity and position according to Equations (19) and (20) until the moving speed of all the particles converges to 0 (less than 10^−6^) or the maximum iteration number is attained.

### 3.2. Joint-PSO Algorithm for SML

From the description above, we can know the following facts of PSO:

(1) Due to the moving mechanism of particles, the closer the initial value of particles are to the optimal value (solution of SML criteria which minimizes $L_{SML}$), the faster all the particles converge to the optimal value.
(2) The search space is the whole solution space of $L_{SML}$. According to the assumptions above, the sources and the sensors are in the same plane. Therefore, the solution space is an area in which each *θ* changes from −90° to 90°.
(3) The PSO algorithm is a population based algorithm. All the particles are randomly initiated in the whole search space.

From the facts above we can see that, in order to guarantee that there are particles close to the optimal value, it usually needs a large number of particles, i.e., *m* should be large enough (usually *m* is not less than 25). However, a large number of particles also bring high computational complexity. Furthermore, since all the particles are randomly initiated in the whole search space, even if there is a particle coincidentally close enough to the optimal value, it still needs a large number of iterations when all the other particles converge to the optimal value. Therefore, for conventional PSO algorithm, too many particles and too many iterations bring high computational complexity.

To overcome these two defects, we will propose a Joint-PSO algorithm which can reduce computational complexity greatly.

#### 3.2.1. A Novel Initialization Space

Firstly, we know that the ESPRIT algorithm can generate the DOA estimation explicitly (for the case where there are coherent signals, the pre-processing techniques should be used [6,7]) and its computational complexity is extremely low. Since in theory the asymptotic covariance matrix of SML is much lower than that of ESPRIT [14,15,16], it means that the estimation accuracy of SML is much higher than that of ESPRIT. But it does not hinder the fact that the solution of ESPRIT (a set of Θ) is close to the solution of SML. Therefore, the solution of ESPRIT should be a rather good initial value of a particle.

Then, to let more particles be close to the solution of SML, we should determine an area which is around the solution of ESPRIT. Let

(21) $${\hat{\Theta }}_{E}=\left\{{\hat{\theta }}_{e1},{\hat{\theta }}_{e2},\ldots ,{\hat{\theta }}_{eq}\right\}$$

represents the solution of ESPRIT. We will use the matrix of stochastic Cramer-Rao Bound, i.e., $CRB_{S}$ together with the solution of ESPRIT to determine the area as the initialization space of our proposed algorithm. The approximate matrix of stochastic Cramer-Rao Bound can be calculated as follow [14,15,16]:

(22) $$CRB_{S}=\;\left[\;\begin{array}{ccc} CRB_{S1} & 0 & 0\\ 0 & \cdots & 0\\ 0 & 0 & CRB_{Sq} \end{array}\;\right]\;=\frac{6}{Np^{3}}\left[\begin{array}{ccc} \frac{1}{SNR_{1}} & 0 & 0\\ 0 & \cdots & 0\\ 0 & 0 & \frac{1}{SNR_{q}} \end{array}\;\right]$$

where *N* is the number of snapshots; *p* is the number of sensors; $SNR_{i},0<i\leq q$ is the SNR of the *i*-th signal. Note that, when the numbers of sensors and snapshots are fixed, the CRB of each source, i.e., $CRB_{Si}$, is uniquely determined by its SNR.

The initialization space can be defined as the range of maximum and minimum values of a set of Θ:

(23) $$\theta_{1}\in \left[{\hat{\theta }}_{e1}-\mu CRB_{S1},{\hat{\theta }}_{e1}+\mu CRB_{S1}\right],$$

(24) $$\theta_{2}\in \left[{\hat{\theta }}_{e2}-\mu CRB_{S2},{\hat{\theta }}_{e2}+\mu CRB_{S2}\right],\ldots$$

(25) $$\theta_{q}\in \left[{\hat{\theta }}_{eq}-\mu CRB_{Sq},{\hat{\theta }}_{eq}+\mu CRB_{Sq}\right],$$

where *μ* is a positive integer. Note that, we choose CRB as a “scale” because CRB is only related with SNR of each source and it is the value of search accuracy with that SNR. Of course, when the sources and the sensors are in the same plane, ${\hat{\theta }}_{ei}-\mu CRB_{Si}\geq$ −90° and ${\hat{\theta }}_{ei}+\mu CRB_{Si}\leq$ 90°.

The size of the initialization space can be controlled by the factor *μ*, a positive integer. Note that when *μ* is very large, for example μ=∞, the initialization space of the proposed algorithm is equal to the original whole search space. In this case, the proposed algorithm is the same to the conventional PSO algorithm.

From the conventional PSO algorithm, we know that large search space usually needs a large number of particles to be covered. Therefore, does it mean that the smaller *μ* is, the fewer particles (*m*) the proposed algorithm needs? We should find the best combination of the values of *μ* and *m* which leads to the lowest computational complexity. We will show the best values of *μ* and *m* from simulation results.

#### 3.2.2. Discussion about the Inertia Factor *w*

In the conventional PSO algorithm, the inertia factor *w* is a fixed value between 0.4 to 0.9. Usually, it is set to be 0.5 [19,20,25]. In our research, we find that when *w* is relatively large between 0.4 to 0.9, the particles move dramatically, when *w* is small between 0.4 to 0.9, the particles move smoothly. Therefore, a large value of *w* between 0.4 to 0.9 is beneficial for the PSO algorithm search the “best” in a larger area, and a small value of *w* between 0.4 to 0.9 is beneficial for fine search in a small area.

For conventional PSO algorithm, in the convergence process, we hope that at the beginning of the search period, the value of *w* should be large between 0.4 to 0.9, so that the algorithm can have a quick speed for updating. At the end of the search period, the value of *w* should be smaller, so that the search result can have higher accuracy. In our previous research [25], we have proposed to change the value of *w* in a parabolic curve according to the iteration times:

(26) $$w\left(l\right)=0.4+\frac{1}{2l_{max}^{2}}{\left(l-l_{max}\right)}^{2},$$

where $l_{max}$ is the maximum iteration number.

For our proposed Joint-PSO algorithm, since the initial particles are already very close the solution of SML, *w* should be a small value between 0.4 to 0.9 so that all the particles can move smoothly to the “best”.

As a result, the value of *w* has an important relationship with the total iteration times and computational complexity. We will show how to determine the best value of *w* from simulation results.

#### 3.2.3. Summary of the Proposed Joint-PSO Algorithm for SML

We summarize the proposed Joint-PSO algorithm for estimation of SML as follows.

(1) Calculate the solution of ESPRIT and the stochastic CRB, then construct a initialization space by Equation (23) to Equation (25).

(2) Randomly initiate a small number of particles $\bar{\Theta }=\left\{{\tilde{\Theta }}_{1},{\tilde{\Theta }}_{2},\ldots ,{\tilde{\Theta }}_{m}\right\}$ in the initialization space. *m* is the number of particles. ${\tilde{\Theta }}_{i}=\left(\theta_{i1},\theta_{i2},\ldots ,\theta_{iq}\right)$ is defined as the *i*th (1≤i≤m) particle’s position in the search space.

(3) Each particle uses the local best position found by itself and the global best position found by the swarm for updating. Let $T_{i}^{l}$ and $G^{l}$ denote the local best and global best of the *l*-th iteration respectively as defined in Equations (17) and (18).

(4) The updating of the particles is accomplished by the following equations:

(27) $${\tilde{\Theta }}_{i}^{l+1}={\tilde{\Theta }}_{i}^{l}+{\tilde{V}}_{i}^{l+1};$$

where,

(28) $${\tilde{V}}_{i}^{l+1}=w{\tilde{V}}_{i}^{l}+c_{1}r_{1}\left(T_{i}^{l}-{\tilde{\Theta }}_{i}^{l}\right)+c_{2}r_{2}\left(G^{l}-{\tilde{\Theta }}_{i}^{l}\right).$$

*l* is the updating times; ${\tilde{V}}_{i}^{0}=0.5$; *w* is the inertia factor; $c_{1}$ is self confidence factor and $c_{2}$ is swarm confidence factor,$c_{1}=c_{2}=2$; $r_{1}$ and $r_{2}$ are uniformly distributed random variables between 0 and 1.

(5) Update velocity and position according to Equations (27) and (28) until the moving speed of all the particles converges to 0 (less than 10^−6^) or the maximum iteration number is attained.

Finally, note that the proposed algorithm can also be applied to many other estimation problems in DOA (multi-dimensional non-linear optimization problem), for example DML, WSF and so on.

## 4. Simulations

In the simulation, firstly we will determine the best values of the important factors *μ*, *m* and *w* by simulation results. Then we will show the efficiency of our proposed algorithm with comparison to the conventional PSO algorithm, classic AM algorithm [9], and Genetic algorithm (GA) [17]. We do simulation using “Matlab” with the version of R2013a in a normal laptop where the CPU is Inter(R) Core(TM) i5-6300U @2.40 GHz and the RAM is 8.0 GB.

In the simulation, the sensors’ geometry is arbitrary. The sensors are randomly located around the reference point. The distances between sensors and the reference point randomly vary from λ/2 to 2λ, where *λ* is the wavelength of signals impinging on the array.

The SNR is defined as:

(29) $${\text{SNR}}_{k}=10\log_{10}\frac{E[{\left|s_{k}\left(t\right)\right|}^{2}]}{\sigma^{2}},$$

where $s_{k}\left(t\right)$ and $\sigma^{2}$ are defined as above. The Root-Mean-Square-Error (RMSE) is defined as

(30) $$\text{RMSE}=\sqrt{\frac{1}{qM}\sum_{k=1}^{q}\sum_{l=1}^{M}{\left|{\hat{\theta }}_{k,l}-\theta_{k}\right|}^{2}},$$

where *M* is the number of independent simulation trails. ${\hat{\theta }}_{k,l}$ is the estimation of $\theta_{k}$ at the *l*-th trial. Therefore RMSE represents the deviation between the estimated value and the ture DOA. The unit is degree. In the simulation, when we compare the computational complexity of each algorithm, the precondition is that the convergence accuracy (convergence condition) should be the same. Otherwise it is not meaningful.

### 4.1. Discussion about the Best Values of μ, m and w

To get the best values of *μ*, *m* and *w*, first of all we have to know the meaning of each parameter. *μ* controls the size of the initialization space, *m* is the number of particles and *w* is the inertia factor. All these parameters are related to the computational complexity. Our purpose is to get the best combination of them which leads to the least computational complexity. Since there are so many parameters, we adopt qualitative analysis combined with quantitative experiment method.

To simplify this problem, firstly we discuss the inertia factor *w* by fixing *μ* and *m*. From Section 3.2.2, we have known that when *w* is relatively large between 0.4 to 0.9, the particles move dramatically, while when *w* is small between 0.4 to 0.9, the particles move smoothly. Therefore, a large value of *w* between 0.4 to 0.9 is beneficial for the PSO algorithm search the “best” in a larger area, and a small value of *w* between 0.4 to 0.9 is beneficial for fine search in a small area. For our proposed Joint-PSO algorithm, since the initialization space is already very close to the solution of SML, it is better for us to choose a small value of w between 0.4 and 0.9. To verify this conclusion, we do simulations with different value of *w* (*w* = 0.4, 0.5, 0.6, ..., 0.9) for fixed *μ* and *m*. Then we select some data as shown in Table 1, Table 2 and Table 3. From Table 1, Table 2 and Table 3, we can find that for fixed *μ* and *m*, when *w* = 0.4, the computational complexity (Cal. time) of the proposed algorithm is much lower than that of other tables. Therefore, for our proposed algorithm, it is better to choose *w* = 0.4.

Next we have to determine the best value of *μ* and *m*. *μ* controls the size of initialization space. If μ=∞, the initialization space is the whole search space. In other words, it is the same to the conventional PSO algorithm. From the characteristics of the PSO algorithm (concluded at the beginning of Section 3.2), we know that for conventional PSO algorithm, to guarantee that there are particles close to the optimal value, *m* should be large enough (usually not fewer than 25). For our proposed Joint-PSO algorithm, since the solution of ESPRIT is already close to the solution of SML, to guarantee that all the particles are close to the solution of SML, it is better to choose a proper value of *μ* (not too large or too small) which can control that the initialization space is not very large and can include the solution of SML. Considering that the stochastic CRB is a small value which is uniquely determined by the number of sensor, snapshots and SNR, the value of *μ* should be not large. Then a small number of particles, i.e., small value of *m*, is necessary. As a result, all the particles can quickly converge to the solution of SML. With these analysis, we select some representative value of *μ* (1, 3, 10, 50 and 500 which mean that the initialization space gradually expands) and *m* (5, 10 and 25) as shown in Table 1, Table 2 and Table 3.

In Table 1, Table 2 and Table 3, the scenario is that p=8, q=2, N = 100, SNR=5 dB, RMSE = 0.26. Two sources are independently located in 30° and −15°. All the cases are done through 30 independent trials. “Cal. time” represents the average calculating time of the algorithm with that value of *μ*, *m* and *w* to find the “best” (solution of SML). The Unit is second. Here the calculating time is the total computational complexity to get the estimation of DOA including the cost of ESPRIT and Stochastic CRB. Note that when μ=∞ (infinity), it means the conventional PSO algorithm is used. We also have to note that for conventional PSO algorithm and the proposed algorithm, the convergence condition is the same, i.e., when the moving speed of all the particles converges to 0 (less than 10^−6^) or the maximum iteration number is attained. The maximum iteration number is 300. Since in the simulation we found that both algorithms can find the global solution of SML successfully and the maximum iteration number is not attained, the RMSE of them are the same as shown in the caption of each Table.

From these Tables, we can find the following two facts. First, our proposed algorithm is much more efficient than the conventional PSO algorithm (μ=∞). Second, for our proposed algorithm, when *w* = 0.4, *μ* = 3, and *m* = 5, the computational complexity is the lowest. Note that, these simulations are just for the case that *p* = 8, *q* = 2 (two independent signals), N = 100, SNR = 5 dB. We have also done simulations for other cases with different SNRs. We find that the best values of *μ* and *m* are not constant. From a large number of simulations, we find that for different cases it is better to choose the value of *μ* between 1 and 10, and *m* between 3 and 10. This is because when *μ* is between 1 and 10, the initialization space is not so large and close enough to the “best” of SML. Since the initialization space is not so large, it is not necessary to set many particles.

As for the effect of different values of ${\tilde{V}}_{i}^{0}$, we also do some simulations and we find that it has little impact on the final computational complexity. That is because the PSO algorithm is an iterative technique and ${\tilde{V}}_{i}^{0}$ is just the moving speed of the first step.

For the conventional PSO algorithm, it is obvious that it is much better to choose the number of particles to be 25 since the whole search space (when μ=∞) is very large. Furthermore, we have discussed the improvement of the value of *w* in [25]. In [25] we proposed to change the value of *w* in a parabolic curve according to (26). The reason is as we have discussed in the Section 3.2.2. For our proposed Joint-PSO algorithm, it is not necessary to make *w* change with iteration times since the number of iteration is usually very small and they are already very close to the solution of SML. In simulations, we find that for our proposed Joint-PSO algorithm, the iteration times are almost the same when w=0.4 and when *w* changes according to Equation (26).

In the simulations below for our proposed Joint-PSO algorithm, we set w=0.4, μ=3, m=5 and for the conventional PSO algorithm, m=25, *w* changing according to Equation (26) if there is no special explanation.

### 4.2. Initialization Space and Convergence of Conventional PSO and Proposed Algorithm

**Figure 1:**
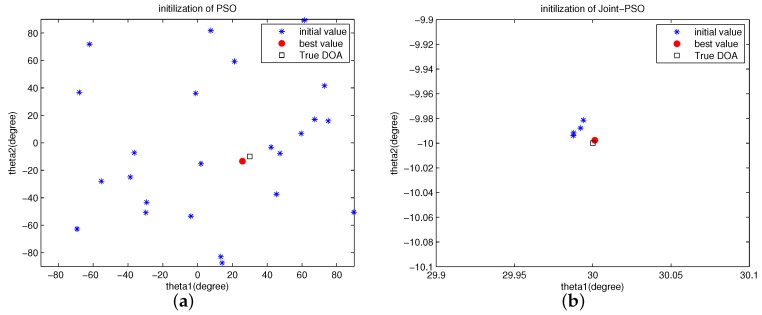
Initialization of conventional Particle Swarm Optimization (PSO) and Joint-PSO algorithms. (**a**) Initialization of PSO algorithm, m=25; (**b**) Initialization of Joint-PSO algorithm, m=5.

In this subsection, we do simulations to show the initialization space and convergence process of the proposed algorithm. In Figures, “Joint-PSO” represents our proposed algorithm since we jointly use the solution of ESPRIT and CRBs. Figure 1 shows the initialization of PSO and Joint-PSO algorithms. The scenario is the same to Table 1, Table 2 and Table 3. “best value” represents the solution of SML. “Ture DOA” is 30° and −15°. The values of *m*, *μ* and *w* are set as above.

**Figure 2:**
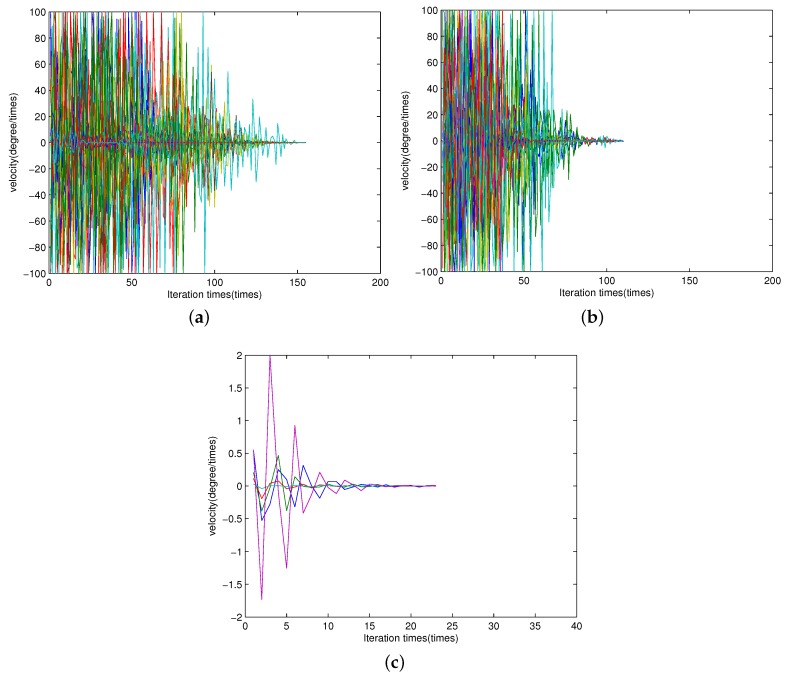
Iteration times of conventional PSO and Joint-PSO algorithms. (**a**) Iteration times of conventional PSO, m=25, w=0.4); (**b**) Iteration times of conventional PSO, m=25, *w* is set according to Equation (26); (**c**) Iteration times of the proposed Joint-PSO, m=5, w=0.4.

In Figure 1, it shows clearly that our proposed Joint-PSO algorithm has a very good initialization space. Since the initialization space is not large and very close to the “best value” (solution of SML), only a small number of particles are necessary (set to be 5) and all the particles are close to the “best value”. As a result, all the particles will converge quickly to the “best value” as shown in Figure 2.

Figure 2 shows samples of the convergence process (moving speed) of all the particles according to the iteration times for PSO and Joint-PSO algorithms. The vertical axis is the velocity of all the particles. The scenario is the same to Figure 1. Figure 2a,b are samples for conventional PSO algorithm with different values of *w*. Figure 2c is a sample for the proposed Joint-PSO algorithm with w=0.4.

The updating process for both algorithms will not be stopped until the moving speed of all the particles converges to 0 (less than 10^−6^) or the maximum iteration number is attained. The maximum iteration number is set to be 300 for conventional PSO algorithm.

From Figure 2a,b, we can find that for conventional PSO algorithm, the number of iteration is reduced about one fifth when *w* is set according to Equation (26). The reason is as we have discussed above. Figure 2c shows that the number of iterations for the proposed Joint-PSO algorithm is much less than that of conventional PSO algorithm. Note that one iteration means an update of all the particles. Since the number of particles of the proposed Joint-PSO algorithm is also much less than that of conventional PSO algorithm, the computational complexity of the proposed algorithm is much lower.

In Figure 2, we just show the moving speed of all the particles of $\theta_{1}$ according to iteration times. The moving speed of the other source ($\theta_{2}$) is almost the same as that of $\theta_{1}$ because the convergence condition is that the moving speed of all the particles converges to 0 (for both $\theta_{1}$ and $\theta_{2}$), or the maximum iteration number is attained. Therefore, we omit the description of $\theta_{2}$.

### 4.3. Comparison of RMSE and Computational Complexity

In this subsection, we will show the efficiency of our proposed Joint-PSO algorithm with comparison to the conventional PSO algorithm, classic AM algorithm [9] and Genetic algorithm (GA) [17]. Note that the original ESPRIT algorithm [3] is formulated based on assumptions that the array is ULA and there is no signal coherent. However, in our simulation, the sensors’ geometry is arbitrary and there are coherent signals. In this case, if we want to use the estimation result of ESPRIT to determine the initialization space, we have to take the following two steps. Firstly, it uses virtual array transformation technique [26] to change the sensors’ geometry to be ULA. Secondly, we need to use the spatial smoothing technique [6] such that it can deal with coherent signals.

In the simulation, for the GA algorithm [17], it also has many parameters such as the population size, the crossover probability and the mutation probability. To have a fair comparison, we also need to choose the best values of them for SML estimation. As a result, we do simulation with different value of each parameter for SML estimation as shown in Table 4. In Table 4, “psize” represents the population size; “mu-prob” represents the mutation probability. “Cal. time” represents the average calculating time, i.e., the whole computational complexity.

From Table 4, we find that with different values of population size and mutation probability, the whole computational complexity changes little. They are in the same order of magnitude. We also do simulations with different values of crossover probability. Similar simulation results are observed. As a result, for GA algorithm, we set that the population size is 60, the crossover probability is 0.6 and the mutation probability is 0.1.

For the AM algorithm [9], in the one-dimensional global search of each updating process, we use different step-size for searching. At first, we have a relatively large step-size (0.1 degree) for rough search and then much smaller step-sizes (0.01, 0.0001 and 10^−6^) for fine search. In this way we can have fewer computation times for one-dimensional global search to attend the convergence accuracy.

Note that since all these algorithms are used to find the global solution of SML criteria. i.e., to solve the multi-dimensional non-linear optimization problem of SML, the convergence accuracy of each algorithm is set to be the same (10^−6^) for identical simulation condition. Therefore, the RMSE of each algorithm for SML estimation is also the same as shown in the caption of each Table. Otherwise, it is meaningless to compare the computational complexity of them.

**Figure 3:**
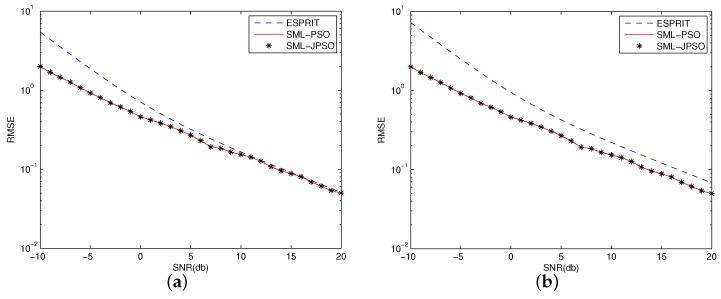
Root-Mean-Square-Error (RMSE) of PSO and Joint-PSO algorithms for Stochastic Maximum Likelihood (SML) and Estimation of Signal Parameters via Rotational Invariance Techniques (ESPRIT). (**a**) Non-coherent case, r=2, two signals are independent; (**b**) Coherent case, r=1, two signals are fully correlated.

The scenario of Figure 3 is that p=8, q=2 and N=100. The ture DOA is 30° and −15°. The “RMSE” is defined as above and the unit is degree. Figure 3 shows the RMSE of PSO and Joint-PSO algorithms for SML with comparison to ESPRIT. From Figure 3a,b, we can find the following facts. First, for both coherent and non-coherent cases, the curve of Joint-PSO coincides with that of PSO and they converge well. It means that both algorithms find the DOA successfully. The RMSE of the other algorithms (AM, GA) also coincides with that of Joint-PSO and conventional PSO algorithms since the SML criteria is the same and their convergence accuracy is also set to be the same. Second, For coherent case, the RMSE of SML, i.e., estimation accuracy, is much better than that of ESPRIT just as we have introduced in the introduction. For non-coherent case, the RMSE of SML coincides with that of ESPRIT when SNR is larger than 10 dB. It means that when SNR is high and there are no coherent signals, it is not necessary to use the SML estimation because the ESPRIT is good enough and its computational complexity is much lower. Therefore, the proposed algorithm for SML estimation is suitable for the cases that when there are coherent signals or when SNR is relatively low.

Next, Let us see the computational complexity of our proposed Joint-PSO algorithm with comparison to the conventional PSO, AM and GA algorithms for different cases as shown in Table 5, Table 6, Table 7 and Table 8. The scenarios are described in the caption of each Table. The label “GA-SML” represents the Gentic algorithm for SML estimation. The other algorithms are labeled in the same manner. Obviously, the computational complexity of the iteration process of each algorithms is determined by “Times of calculation of $L_{SML}$” which are the production of “Number of particles” and “Average iteration times”, while “Total calculating time” represents the whole computational complexity including the calculation of ESPRIT and CRB. Note that when there are coherent signals, the cost of the pre-processing techniques are also included.

Table 5 shows the non-coherent case; Table 6 shows the coherent case; Table 7 shows the case that two signals are very close, while Table 8 shows the case that there are 4 independent signals. From all these tables, we can find that the proposed Joint-PSO algorithm is the most efficient. It is about one-tenth of the conventional PSO algorithm and almost one percent of GA and AM algorithms. Similar simulation results are observed in many other cases. These simulations prove the great efficiency of the proposed algorithm.

Note that, in Table 5, the estimation of ESPRIT takes only 0.0015 s (in Table 6 ESPRIT takes 0.0023 s; in Table 7 ESPRIT takes 0.0021 s; in Table 8 ESPRIT takes 0.0042 s) which is about one-tenth of the time our proposed algorithm takes. That is because the estimation of ESPRIT is not a multi-dimensional non-linear optimization problem and it can be calculated explicitly. The defect of ESPRIT is that its accuracy is much lower than that of SML as shown in Figure 3. However, the ESPRIT is still one of the most commonly used algorithms in real systems because of its low computational complexity. On the other hand, from Table 5, Table 6, Table 7 and Table 8 we can find that for SML estimation our proposed algorithm is the most efficient one among all the solving techniques until now. Furthermore, we should note that the calculating time of the proposed Joint-PSO algorithm could even be greatly reduced if the parallel computing and distributed computing techniques are used. In these cases, the calculating time of our proposed algorithm for SML estimation could be comparable to that of ESPRIT. Therefore, our proposed Joint-PSO algorithm shows great potential for the application of SML in real systems.

## 5. Conclusions

In this paper, to reduce the computational complexity of SML estimation we proposed a simple but effective modification of conventional PSO algorithm. The proposed Joint-PSO algorithm uses the solution of ESPRIT and stochastic CRB to determine a novel initialization space which is close to the solution of SML. Then fewer particles and iteration times are needed. As a result, computational complexity can be reduced greatly. The value of our proposed Joint-PSO algorithm is as follows. First, the proposed Joint-PSO algorithm is general. It can be applied to some other multi-dimensional non-linear optimization problems of estimation of DOA such as DML and WSF. Second, the proposed algorithm is one of the most efficient solving techniques for SML estimation and it shows great potential for the application of SML in real systems.

## Figures and Tables

**Table 1:** Average calculating time with different *μ* and *m* where w=0.4, p=8, q=2, N = 100, Signal-to-Noise Ratio (SNR) = 5 dB, Root-Mean-Square-Error (RMSE) = 0.26.

	Cal. Time	*μ*	*μ* = 1	*μ* = 3	*μ* = 10	*μ* = 50	*μ* = 500	*μ* = Infinity (Conv. PSO)
*m*								
*m* = 5			0.036	0.019	0.038	0.047	0.072	0.543
*m* = 10			0.042	0.038	0.043	0.068	0.087	0.41
*m* = 25			0.054	0.053	0.087	0.097	0.158	0.358

**Table 2:** Average calculating time with different *μ* and *m* where w=0.6, p=8, q=2, N = 100, SNR=5 dB, RMSE = 0.26.

	Cal. Time	*μ*	*μ* = 1	*μ* = 3	*μ* = 10	*μ* = 50	*μ* = 500	*μ* = Infinity (Conv. PSO)
*m*								
*m* = 5			0.036	0.039	0.042	0.053	0.073	0.65
*m* = 10			0.046	0.068	0.083	0.098	0.12	0.53
*m* = 25			0.065	0.096	0.11	0.144	0.186	0.48

**Table 3:** Average calculating time with different *μ* and *m* where w=0.8, p=8, q=2, N = 100, SNR=5 dB, RMSE = 0.26.

	Cal. Time	*μ*	*μ* = 1	*μ* = 3	*μ* = 10	*μ* = 50	*μ* = 500	*μ* = Infinity (Conv. PSO)
*m*								
*m* = 5			0.052	0.052	0.063	0.065	0.075	0.672
*m* = 10			0.089	0.092	0.105	0.124	0.156	0.42
*m* = 25			0.159	0.162	0.164	0.181	0.21	0.36

**Table 4:** Genetic algorithm (GA) for SML estimation with different parameters where crossover probability is 0.5, p=8, q=2,r=2, N = 100, SNR=5 dB, 30 independent trials, RMSE = 0.26.

	Cal. Time	mu-prob	mu-prob = 0.2	mu-prob = 0.1	mu-prob = 0.01
Psize					
*psize* = 50			2.81	2.76	2.60
*psize* = 100			2.72	2.86	2.45
*psize* = 200			2.54	2.65	2.33
*psize* = 500			2.63	2.83	2.58

**Table 5:** Comparison of computational complexity of Joint-PSO, PSO, GA and AM for SML. Scenario: p=8,q=2,r=2(non−coherent),N=100,SNR=0 dB, The ture DOA: 30° and −15°, 30 independent trials, RMSE = 0.45.

	Joint-PSO-SML	PSO-SML	GA-SML	AM-SML
Number of particles	5	25	–	–
Average iteration times	23.5	107.9	–	–
Times of calculation of $L_{SML}$	5 × 23.5 = 117.5	25 × 107.9 = 2697.5	–	–
Total calculating time (second)	0.019	0.198	2.33	3.64

**Table 6:** Comparison of computational complexity of Joint-PSO, PSO, GA and AM for SML. Scenario: p=8,q=2,r=1(coherent),N=100,SNR=0 dB, The ture DOA: 30° and 10^−6^, 30 independent trials, RMSE = 0.53.

	Joint-PSO-SML	PSO-SML	GA-SML	AM-SML
Number of particles	5	25	–	–
Average iteration times	24	110	–	–
Times of calculation of $L_{SML}$	5 × 24 = 120	25 × 110 = 2750	–	–
Total calculating time (second)	0.02	0.204	2.36	3.67

**Table 7:** Comparison of computational complexity of Joint-PSO, PSO, GA and AM for SML. Scenario: p=8,q=2,r=2,N=100,SNR=0 dB, The ture DOA: 30° and 29.5° (close sources), 30 independent trials, RMSE = 0.45.

	Joint-PSO-SML	PSO-SML	GA-SML	AM-SML
Number of particles	5	25	–	–
Average iteration times	28.7	121.3	–	–
Times of calculation of $L_{SML}$	5 × 28.7 = 143.5	25 × 121.3 = 3032.5	–	–
Total calculating time (second)	0.023	0.221	2.39	3.71

**Table 8:** Comparison of computational complexity of Joint-PSO, PSO, GA and AM for SML. Scenario: p=8,q=4,r=4,N=100,SNR=0 dB, The ture DOA: 10°, 20°, 30° and 40° (4 sources), 30 independent trials, RMSE = 0.48.

	Joint-PSO-SML	PSO-SML	GA-SML	AM-SML
Number of particles	5	25	–	–
Average iteration times	41.3	143.5	–	–
Times of calculation of $L_{SML}$	5 × 41.3 = 206.5	25 × 143.5 = 3587.5	–	–
Total calculating time (second)	0.043	0.429	4.53	6.58
